# Supplementary material for: The docking of synaptic vesicles on the presynaptic membrane induced by α-synuclein is modulated by lipid composition
Source: Nat Commun. 2021 Feb 10;12:927. doi: 10.1038/s41467-021-21027-4 (PMC7876145; doi:10.1038/s41467-021-21027-4)
Supplement: Supplementary file 1 — Supplementary Information [file 41467_2021_21027_MOESM1_ESM.pdf]

Supplementary Material

The Docking of Synaptic Vesicles on the Presynaptic Membrane Induced by α-Synuclein is Modulated by Lipid Composition

Wing K. Man[1], Bogachan Tahirbegi[2], Michail Vrettas[3], Swapan Preet[1], Liming Ying[4], Michele Vendruscolo[1], Alfonso De Simone[3,5,*], Giuliana Fusco[1,*]

[1]*Centre for Misfolding Diseases, Department of Chemistry, University of Cambridge, Cambridge CB2 1EW UK*

[2]Department of Chemistry, Imperial College London, Molecular, Sciences Research Hub, Wood Lane, W12 0BZ UK.

[3]*Department of Pharmacy, University of Naples "Federico II", Naples IT*

[4]*National Heart and Lung Institute, London Wood Lane, W12 0BZ UK*

[5]*Department of Life Sciences, Imperial College London, South Kensington, SW7 2AX UK*

*Correspondence: adesimon@imperial.ac.uk and gf203@cam.ac.uk

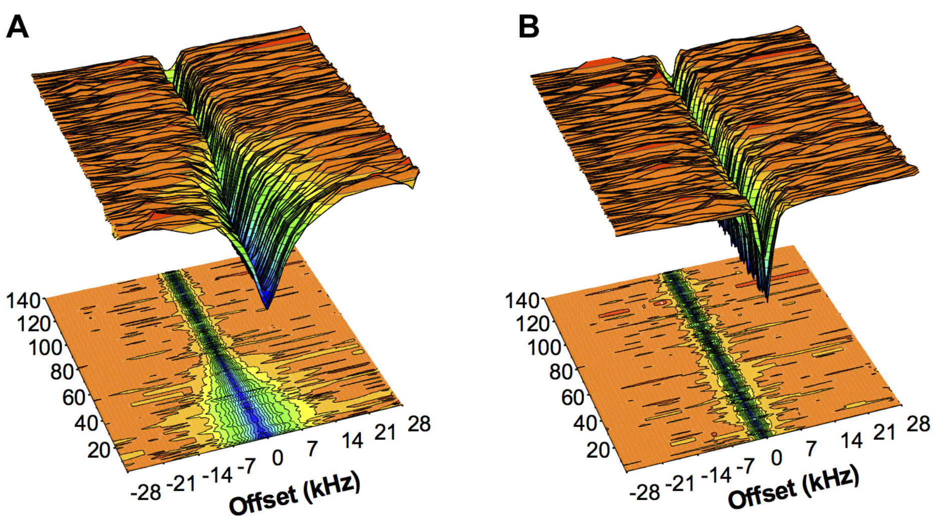

Figure S1 | NMR CEST surfaces of the binding of αS to IPM and OPM measured using a saturation bandwidth of 350 Hz. The surfaces probed the interaction between αS and IPM (A) and OPM (B), and report the saturations along the αS sequence and in a range of offsets (−28, −21, −14, −9, −5, −3, −1.5, 0, 1.5, 3, 5, 9, 14, 21, and 28 kHz). The reference spectrum was measured using a saturation of –100 kHz.

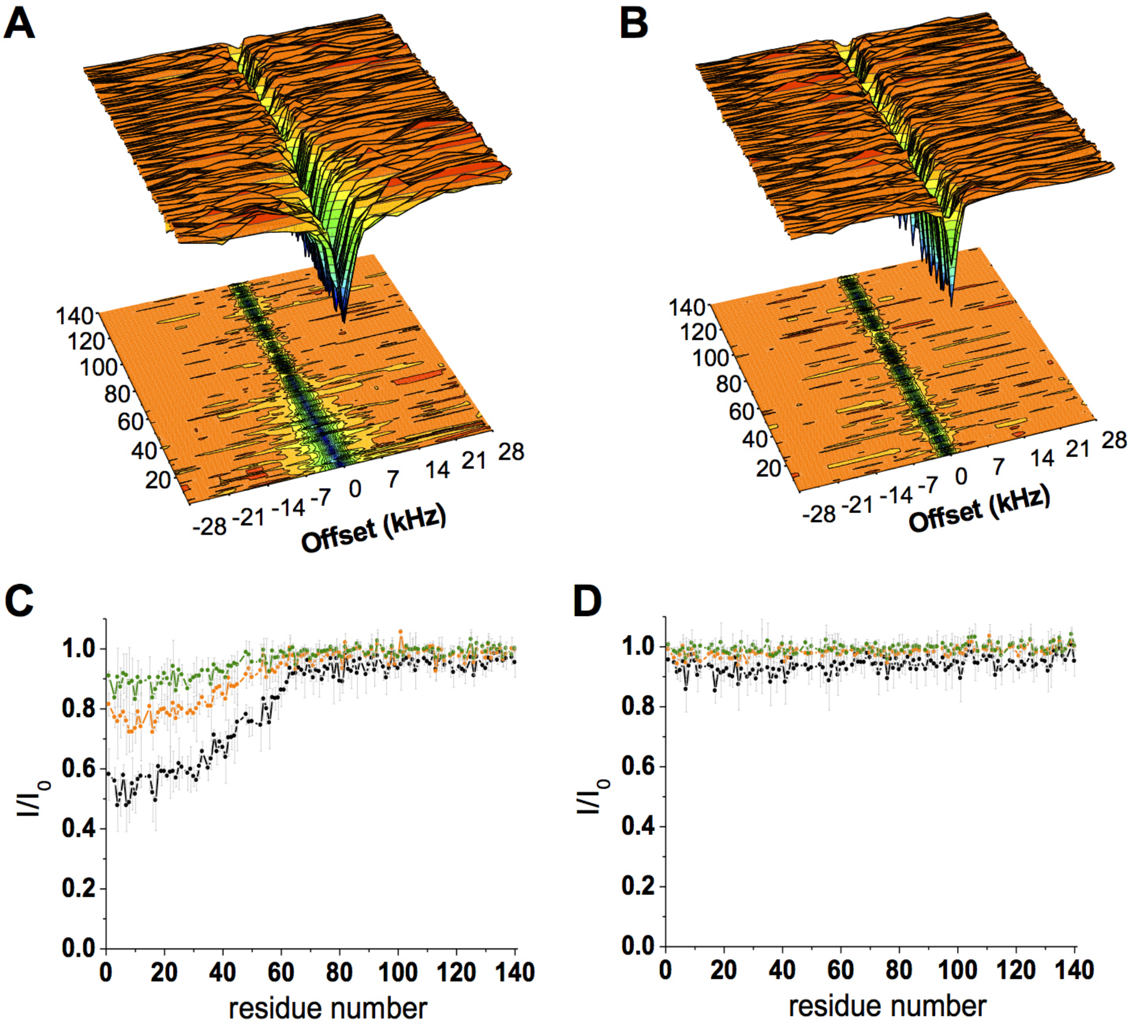

Figure S2 | NMR CEST experiments of the binding of αS to IPM and OPM measured using a saturation bandwidth of 170 Hz. (A,B) NMR CEST surfaces are shown for the interaction between αS and IPM (A) and OPM (B). The surfaces report the saturations along the αS sequence and in a range of offsets (−28, −21, −14, −9, −5, −3, −1.5, 0, 1.5, 3, 5, 9, 14, 21, and 28 kHz). Reference spectrum was measured using a saturation of –100 kHz. **(C,D)** NMR CEST profiles measured using a saturation bandwidth of 170 Hz along the sequence. Interaction of αS with IPM and OPM are reported in panels, C and D respectively. Black, orange and green lines refer to the averaged CEST profiles measured using offsets at ± 1.5 kHz, ± 3.0 kHz, and ± 5.0 kHz, respectively. Error bars report the standard deviation estimated on the triplicate measurements. Source data for this panels C-D are provided as a Source Data file.

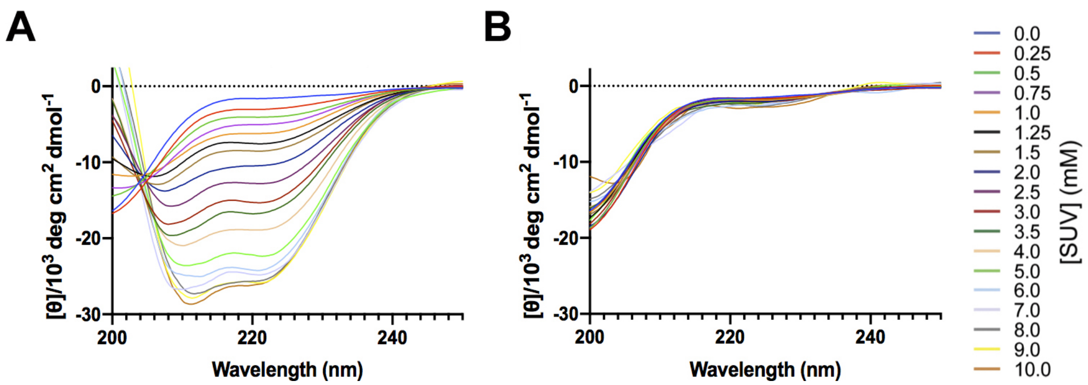

Figure S3 | Interaction between αS and IPM and OPM monitored by circular dichroism (CD). CD profiles were measured using a fixed concentration of αS (10 μM) and variable amounts of IPM **(A)** and OPM **(B)** SUVs, at 283 K in 20 mM of phosphate buffer at pH 6.0. The incubation of αS with IPM SUVs induced the typical α-helical signal being detected CD measurements, particularly at high concentrations of the vesicles. By contrast, no significant perturbation of the random-coin CD profile of isolated αS was detected in CD measurements in the presence of OPM, including conditions in which the protein:lipid ratio was set to 1:1000.

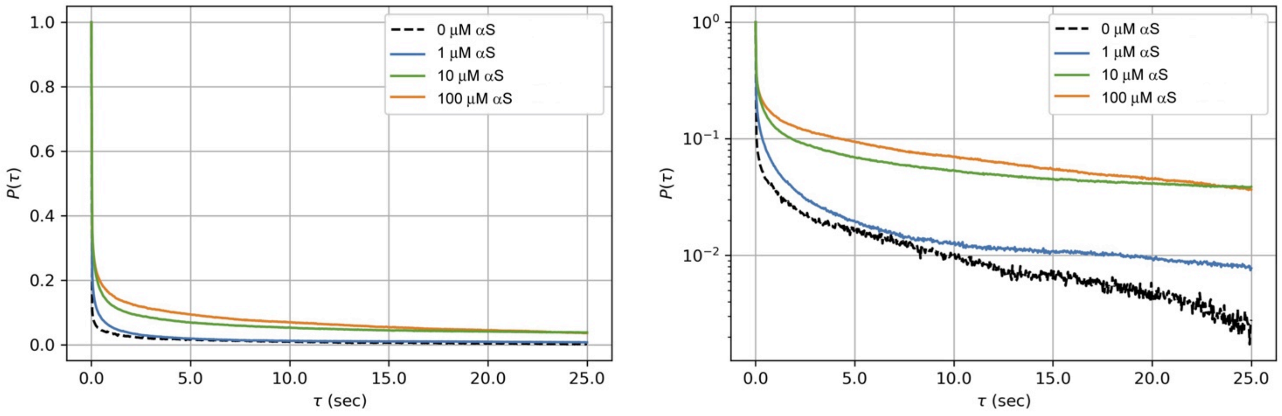

Figure S4 | Residence time of SL-SUVs on IPM surfaces as monitored by TIRF imaging. The residence time was calculated using an autocorrelation function based on the images of SL-SUVs in the TIRF focal plane over time. Autocorrelation functions were then fitted using a double exponential decay (Methods) with the slow component accounting for the residence time of the vesicles in their docking position. The plots show the decrease of the decay of the autocorrelation curve as a function of the concentration of αS. Linear (left) and logarithmic (right) scales are shown in this figure.

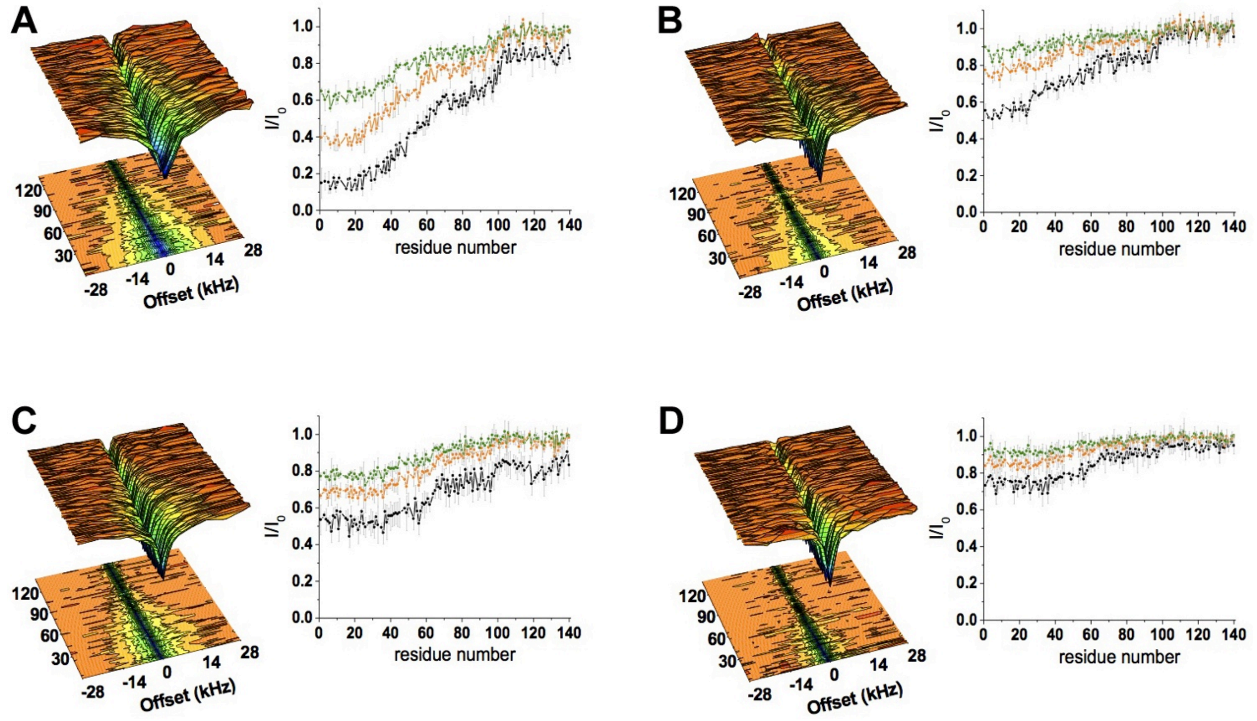

Figure S5 | NMR CEST experiments of the binding of αS to IPM-GMs and OPM-GMs. NMR CEST surfaces, measured using a saturation bandwidth of 350 Hz (A, C) and 170 Hz (B, D), are shown for the interaction between αS and IPM-GMs (A, B) and OPM-GMs (C, D). The surfaces (left side in each panel) report the saturations along the αS sequence and in a range of offsets (−28, −21, −14, −9, −5, −3, −1.5, 0, 1.5, 3, 5, 9, 14, 21, and 28 kHz). Reference spectrum was measured using a saturation of –100 kHz. NMR CEST profiles along the sequence are shown on the right in each panel. Black, orange and green lines refer to the averaged CEST profiles measured using offsets at ± 1.5 kHz, ± 3.0 kHz, and ± 5.0 kHz, respectively. Error bars report the standard deviation estimated on the triplicate measurements. Source data for this panels A-D are provided as a Source Data file.
