# Supplementary material for: The docking of synaptic vesicles on the presynaptic membrane induced by α-synuclein is modulated by lipid composition
Source: Nat Commun. 2021 Feb 10;12:927. doi: 10.1038/s41467-021-21027-4 (PMC7876145; doi:10.1038/s41467-021-21027-4)
Supplement: Supplementary file 3 — Reporting Summary [file 41467_2021_21027_MOESM3_ESM.pdf]

nature research

Corresponding author(s): Alfonso De Simone and Giuliana Fusco

Last updated by author(s): 22/12/2020

Reporting Summary

Nature Research wishes to improve the reproducibility of the work that we publish. This form provides structure for consistency and transparency in reporting. For further information on Nature Research policies, see our Editorial Policies and the Editorial Policy Checklist.

Statistics

For all statistical analyses, confirm that the following items are present in the figure legend, table legend, main text, or Methods section.

n/a	Confirmed	
☐	☒	The exact sample size (n) for each experimental group/condition, given as a discrete number and unit of measurement
☐	☒	A statement on whether measurements were taken from distinct samples or whether the same sample was measured repeatedly
☐	☒	The statistical test(s) used AND whether they are one- or two-sided<br>*Only common tests should be described solely by name; describe more complex techniques in the Methods section.*
☒	☐	A description of all covariates tested
☒	☐	A description of any assumptions or corrections, such as tests of normality and adjustment for multiple comparisons
☐	☒	A full description of the statistical parameters including central tendency (e.g. means) or other basic estimates (e.g. regression coefficient) AND variation (e.g. standard deviation) or associated estimates of uncertainty (e.g. confidence intervals)
☐	☒	For null hypothesis testing, the test statistic (e.g. F, t, r) with confidence intervals, effect sizes, degrees of freedom and P value noted<br>*Give P values as exact values whenever suitable.*
☒	☐	For Bayesian analysis, information on the choice of priors and Markov chain Monte Carlo settings
☒	☐	For hierarchical and complex designs, identification of the appropriate level for tests and full reporting of outcomes
☒	☐	Estimates of effect sizes (e.g. Cohen's d, Pearson's r), indicating how they were calculated

Our web collection on statistics for biologists contains articles on many of the points above.

Software and code

Policy information about availability of computer code

Data collection	Topspin 3.6.0 (Bruker, AXS GmBH, DE)<br>Spectra Manager™ 2.8 (JASCO Research Ltd. CA)<br>HCImage 4.6.1 (Hamamatsu Photonics, JP)
Data analysis	NMRpipe 10.9 (Delaglio et al J. Biomol. NMR. 6, 277-293 (1995). University of Maryland, USA).<br>Sparky 3.1 (Goddard TD & Kneller DG (2008) SPARKY 3. University of California San Francisco, USA)<br>GraphPad Prism 9 (GraphPad Software, USA)<br>ImageJ 1.52p (National Institute of Health, USA)

For manuscripts utilizing custom algorithms or software that are central to the research but not yet described in published literature, software must be made available to editors and reviewers. We strongly encourage code deposition in a community repository (e.g. GitHub). See the Nature Research guidelines for submitting code & software for further information.

Data

Policy information about availability of data

All manuscripts must include a data availability statement. This statement should provide the following information, where applicable:

- Accession codes, unique identifiers, or web links for publicly available datasets
- A list of figures that have associated raw data
- A description of any restrictions on data availability

Data supporting the findings of this study are available within the article and its Supplementary Information Files and from the corresponding author on request. Python processing scripts can be found at https://github.com/vrettasm/analysis_code_Man_et_al

Field-specific reporting

Please select the one below that is the best fit for your research. If you are not sure, read the appropriate sections before making your selection.

- [x] Life sciences
- [ ] Behavioural & social sciences
- [ ] Ecological, evolutionary & environmental sciences

For a reference copy of the document with all sections, see nature.com/documents/nr-reporting-summary-flat.pdf

Life sciences study design

All studies must disclose on these points even when the disclosure is negative.

Sample size	The study presented in this manuscript does not comprise sample size and no statistical methods were used to predetermine the sample size. All molecules presented in the test tubes have been collectively probed in bulk measurements.
Data exclusions	No data were excluded from the study
Replication	The NMR data presented derive from a data collection based on one sample replicate and three experimental replicates. The CD data presented derive from a data collection based on one sample replicate and ten experimental replicates. CD experiments were repeated three times, using completely independent - freshly prepared - samples and resulting in reproducible results. The TIRF data presented derive from a data collection based on one sample replicate and three experimental replicates.<br>For these techniques, in addition to the experimental replicates reported in the manuscript, additional sample replicates for CEST NMR, CD and TIRF imaging (one each) were prepared, providing extremely similar results to those reported in the article.
Randomization	Sample were not randomised for this study due to the small number of specialised samples that were prepared
Blinding	Sample blinding was not relevant given the ability of NMR to identify the sample from the spectrum and of TIRF to identify fluorescently labelled vesicles from the images

Reporting for specific materials, systems and methods

We require information from authors about some types of materials, experimental systems and methods used in many studies. Here, indicate whether each material, system or method listed is relevant to your study. If you are not sure if a list item applies to your research, read the appropriate section before selecting a response.

Materials & experimental systems

n/a	Involved in the study	
x		Antibodies
x		Eukaryotic cell lines
x		Palaeontology and archaeology
x		Animals and other organisms
x		Human research participants
x		Clinical data
x		Dual use research of concern

Methods

n/a	Involved in the study	
x		ChIP-seq
x		Flow cytometry
x		MRI-based neuroimaging
